# Editorial: Reviews in psychology of language-volume II

**DOI:** 10.3389/fpsyg.2026.1909048

**Published:** 2026-07-07

**Authors:** Antonio Benítez-Burraco, Antonio Bova, Thomas Spalding

**Affiliations:** 1 Department of Spanish, Linguistics, and Theory of Literature, Faculty of Philology, University of Seville, Seville, Spain; 2 Department of Psychology, Faculty of Psychology, Università Cattolica del Sacro Cuore, Milan, Italy; 3 Department of Psychology, Faculty of Arts, University of Alberta, Edmonton, AB, Canada

**Keywords:** clinical linguistics, first language acquisition, language teaching, psycholinguistics, second language learning

Over the last few decades, psycholinguistics has crucially contributed, together with neurolinguistics, to our understanding of how our brain processes language. Although languages are sensitive to their speakers' physical and social environment, the core features of language and how they are used depend primarily on how our brain is configured and works. Significant progress has been made in recent years in our understanding of language comprehension and production, language use in natural settings, language acquisition in children and language learning in adults, and language change throughout the lifespan. These are still key areas of interest for psycholinguistics, particularly now that they are being increasingly cross-fertilized by other related disciplines: neurolinguistics, as noted, but also biolinguistics, neuroscience, anthropology, and cognitive sciences, to name a few. At the same time, advances in many fields have opened up new avenues of research that practitioners of psycholinguistics and related fields should be aware of.

As with its first edition, the aim of this Research Topic is to gather comprehensive and up-to-date review articles on key aspects of psycholinguistics, with a focus on the topics and methodologies that are currently in the spotlight, such as the use of artificial intelligence for the improvement of our understanding of language processing by the brain or our teaching methods, or the role of linguistic and cognitive diversity in refining our understanding of language psychology. In this second volume of the Research Topic “*Reviews in Psychology of Language*,” we have collected 10 contributions by 21 scholars.

Two articles address fundamental, traditional concerns of psycholinguistics. The contribution by Pu et al. re-examines the links between language and cognition. The authors support an active role for language in structuring our minds. They focus on the effect of language on executive function and social cognition. Adopting a multidisciplinary approach, they draw on evidence from behavioral, developmental, clinical, and neuroscience studies to propose a more integrative and dynamic view of the place of language within our cognitive architecture. The contribution by Mangat et al. focuses on what is perhaps the most familiar effect in psycholinguistics: semantic priming. The authors review the sources (automatic or strategic) of this effect and propose ways to improve current models of semantic priming.

The majority of articles in this Research Topic examine first language (L1) acquisition in children or second language (L2) learning in more or less formal contexts. Specifically, three articles focus on language acquisition by children. The contribution by Zhang et al. is a meta-analysis of approximately 100 studies examining whether the expression of emotional content by parents has a positive impact on the oral narrative abilities of their children. The authors conclude that the available evidence supports both direct and indirect effects. In the same vein, the article by Smith and Glenwright reviews the diverse research methods used for the determination of the impact of intonation on children's ability to understand verbal irony. The article also comments on the cues used by children to identify verbal irony, along with the cross-linguistic factors impacting this pragmatic use of intonation. Finally, the contribution by Noh et al. aims to characterize the different methodologies used for the evaluation of reading abilities in youth vs. adults. These authors review approximately 50 studies and conclude that adults are typically evaluated in terms of a broader range of skills and abilities and suggest some methodological strategies to overcome current limitations in analyzing reading abilities in children.

Two studies in this Research Topic compare aspects of language acquisition by L1 and L2 learners. The contribution by Polo and Marzi also focuses on reading abilities. More precisely, these authors introduce finger tracking as a new method of gaining insight into the predictive and integrative mechanisms involved in real-time language comprehension during reading. They apply this method to examine reading behavior by native speakers and learners of English. Similarly, the article by Zhu adopts a corpus approach to examine the differential sensitivity of native speakers and L2 learners of English to phonetic issues, particularly the consonant contexts in which vowels appear. Two other studies focus specifically on (methodological) aspects of second language learning. The contribution by Yu reviews the positive and negative impacts of technology on reducing the anxiety experienced by L2 learners of Chinese and includes some methodological recommendations for future research on this topic. In line with this contribution, the article by Yu reviews the potential of positive psychology for the improvement of L2 learning by reducing learning anxiety.

Finally, the contribution by Imaezue et al. is a clinical linguistics study that urges the adoption of more inclusive ways of treating aphasia, particularly those that are more sensitive to linguistic diversity.

